# The Burden of Hypertension and Kidney Disease in Northeast India: The Institute for Indian Mother and Child Noncommunicable Diseases Project

**DOI:** 10.1155/2014/320869

**Published:** 2014-01-27

**Authors:** Maurizio Gallieni, Angela Aiello, Benedetta Tucci, Valeria Sala, Sujit K. Brahmochary Mandal, Anna Doneda, Simonetta Genovesi

**Affiliations:** ^1^Nephrology and Dialysis Unit, San Carlo Borromeo Hospital, Graduate School of Nephrology, University of Milan, Via Pio II, 3-20153 Milano, Italy; ^2^Dialysis Unit, I.R.C.C.S. Policlinico San Donato, 20097 San Donato Milanese, Italy; ^3^Nephrology and Dialysis Unit, San Gerardo Hospital, Graduate School of Nephrology, University of Milano Bicocca, 20090 Monza, Italy; ^4^Institute for Indian Mother and Child, Teghoria, Sonarpur, Kolkata, West Bengal 700150, India; ^5^Project for People, 20125 Milan, Italy

## Abstract

Chronic noncommunicable diseases (NCDs) such as hypertension, atherosclerosis, acute myocardial infarction, stroke, diabetes, obesity, and chronic kidney disease are the major cause of death not only in high income, but also in medium and low income countries. Hypertension and diabetes, the most common causes of chronic kidney disease, are particularly common in southeast Asian Countries. Because early intervention can markedly slow the progression of these two killer diseases, assessment of their presence through screening and intervention program is a priority. We summarize here results of the screening activities and the perspectives of a noncommunicable diseases project started in West Bengal, India, in collaboration with the Institute for Indian Mother and Child (IIMC), a nongovernmental voluntary organization committed to promoting child and maternal health. We started investigating hypertension and chronic kidney disease with screen in school-age children and in adults >30 years old. We found a remarkable prevalence of hypertension, even in underweight subjects, in both children and adult populations. A glomerular filtration rate <60 mL/min was found in 4.1% of adult subjects significantly higher than that of 0.8% to 1.4% reported 10 years ago. Increased awareness and intervention projects to identify NCDs and block their progression are necessary in all countries.

## 1. Introduction

In high income countries, the high prevalence of and the morbidity and mortality from chronic noncommunicable diseases (NCDs) such as hypertension, atherosclerosis, acute myocardial infarction, stroke, diabetes, obesity, and chronic kidney disease are well known [1], and many efforts are taking place for the primary prevention of NCD. The situation is different in low to medium income countries in which, in the past few decades, attention was focused on communicable diseases. Although the battle against communicable diseases is still open and needs to be adequately supported, the improvement of sanitary and living conditions as well as medical advances in terms of vaccination and antibiotic therapy has successfully reduced infant mortality and deaths from uncontrolled epidemics. There is still a great need of intervention in the fight against communicable diseases as demonstrated, for example, by the recent polio outbreaks in Africa and Pakistan, representing a serious setback in the global effort to eradicate polio [2]. However, on the other hand, adopting the lifestyle of industrialized nations, such as decreased physical activity and increased intake of dietary fats and unhealthy diet, and the increase in average life expectancy have resulted in the emergence of noncommunicable diseases as major problems in low income countries [3, 4]. In this setting, achieving primary prevention projects may be difficult, because of limited data on the prevalence of NCD as well as for the great differences in health conditions and in the possibility of access to medical care between different geographical areas, gender, and social stratification.

Ischemic heart disease is the main cause of death in adults from both low and middle income countries as well as from high income countries [5]. Southeast Asia especially faces an epidemic of chronic noncommunicable diseases, now responsible for 60% of deaths in the region [4]. Indonesia (a middle income country) had one of the highest mean levels of systolic blood pressure whereas Brunei (a high income country) had values close to the average for the region. For total cholesterol, the highest mean concentrations were seen in Vietnam, a low income country [4]. Diabetes is increasing in many Asian countries much more than elsewhere. Between 1970 and 2005, the prevalence of diabetes quadrupled in Indonesia, Thailand, India, and China compared with an increase of only 1.5 times in the USA [6].

Chronic kidney disease (CKD) is another NCD which has an increasing importance. It is strongly linked to cardiovascular morbidity for traditional cardiovascular risk factors and “nontraditional” factors, such as fluid overload, anemia, inflammation, metabolic abnormalities, malnutrition, and hyperphosphatemia. The principal causes of chronic kidney disease in low income countries are chronic glomerulonephritis and interstitial nephritis reflecting the high prevalence of bacterial, viral, and parasitic infections that can affect the kidneys. However, diabetes causes 9.1 to 29.9 percent of the cases of end stage renal disease, and hypertension leads to 13 to 21 percent of the cases in the same countries [7]. In Pakistan, Jafar [8] reports 15 to 20 percent of persons 40 years of age or older having a reduced estimated GFR. Although GFR estimation equations have not been validated in Asian populations, such a burden is consistent with the high Asian prevalence of diabetes and hypertension, the two main risk factors for chronic kidney disease.

The prevalence of type 2 diabetes in countries of the southeast Asian region is high. It has been reported that a significant epidemic of diabetes is present, with a rapid increase in prevalence over the last two decades and a projection of further marked increase over the next two decades [9]. Also the burden of hypertension is even higher, affecting approximately 35% of the adult population in the region and accounting for nearly 1.5 million deaths (9.4%) annually [10].

CKD develops in about third of patients with diabetes, and while in people with treated essential hypertension CKD is present in a small percentage of patients [11], the number of hypertensive patients is so large that even the small percentage at risk constitutes a significant portion of those developing CKD. Untreated patients developing severe hypertension have an adjusted relative risk of developing ESRD of 11.2 compared to normotensive patients [11]. Most subjects in southeast Asian region are still not tested and/or treated for hypertension.

Singh et al. [12] in North India found a prevalence of low eGFR (<60 mL/min/1.73  m^2^) of 13.3% by the Cockcroft-Gault (CG) equation and 4.2% by the modification of diet in renal disease (MDRD) equation. Only 3.3% of subjects with renal impairment were aware of their disease.

The cost of treatment of NCD can be very high, in some cases unaffordable for many, as is the case for ESRD. Disability and work disability resulting from NCD are also a heavy burden on societies making a strategy of prevention of this group of diseases a priority. Screening and intervention can prevent chronic kidney disease, and where management strategies have been implemented, the incidence of end stage kidney disease has been reduced [13]. Timely recognition and treatment of diabetes, hypertension, and chronic kidney disease, as well as the institution of preventive measures to control their expansion, are a critical issue, especially in low income countries [14]. Action is needed, and remarkable initiatives are already ongoing, such as the constitution of the NCD alliance (http://www.ncdalliance.org/). On the NCD website, it is reported that “the rapidly changing health and disease profile has serious implications for poverty reduction and economic development. NCDs strangle macro-economic development and keep the bottom billion of the world population locked up in chronic poverty. NCDs have a severe impact on individuals, communities and countries. The magnitude and rapid spread of NCDs means we are all headed for a sick future unless we take action now. Low income countries still grappling with heavy burdens of infectious disease risk being overwhelmed by this wave of largely preventable NCDs.”

**Figure 1:**
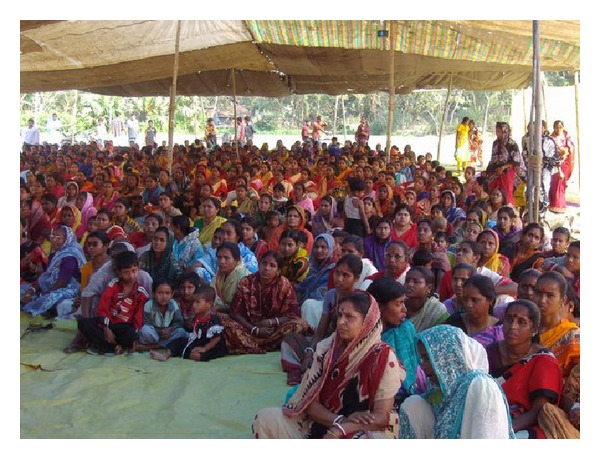
Educational activities on nutrition for mothers.

**Figure 2:**
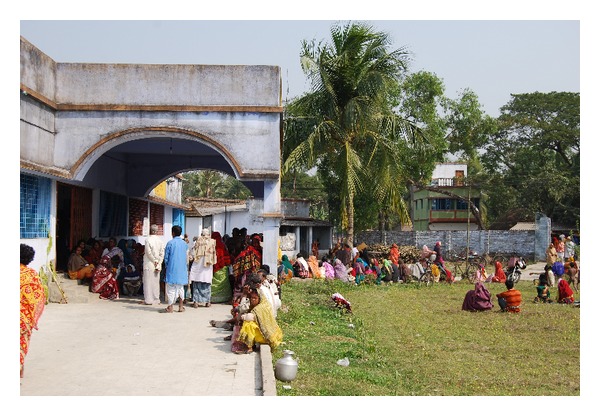
A very busy outdoor clinic in Dhaki, a rural village located in the Ganges river delta, south of Kolkata, India.

In this paper, we report the NCD project developed in the south area of Kolkata, India, through a strict collaboration with the local Institute for Indian Mother and Child (IIMC), a nongovernmental voluntary organization, committed to promote child and maternal health and literacy and to accelerate international solidarity and peace (http://www.iimcmissioncal.org/). Founded in 1989, today IIMC is working with a small 20-bed hospital, an education centre, a women's cooperative unit, and a mother's bank and microfinance unit for poor mothers. The IIMC conducts its various educational (Figure 1), medical, health, and economical uplifting activities in the surrounding remote villages of South 24 Parganas as well as in West Bengal. Currently, IIMC operates 5 outdoor clinics (Figure 2), 5 microfinance banks, 23 schools, and 2 agriculture projects.

**Figure 3:**
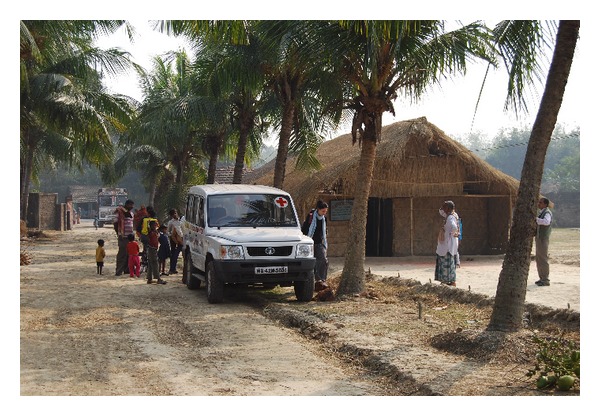
Screening day for noncommunicable diseases in a rural village. The screening took place in the local school (on the right side of the figure), managed by IIMC (Institute for Indian Mother and Child).

Few data are available on the prevalence of NCDs in West Bengal. Therefore, in 2007, we started a project investigating hypertension and chronic kidney disease in the area already covered by IIMC interventions. The goal of IIMC is to improve the overall status of the poorest and weakest through basic interventions in education, social support, and creating the opportunities of employment and self-employment. Medical care is also a major issue focusing on preventive medicine. In this context, we conducted a series of studies on the population of West Bengal and found novel and relevant data regarding hypertension and its relationship with body weight indicators, proteinuria, and eGFR [15, 16]. We discuss here the overall status of the project outlining possible future interventions. We measured arterial blood pressure and other clinical parameters (urinalysis, plasma creatinine, glucose, and proteinuria) as well as anthropometric parameters in children and adults (Figure 3).

## 2. Prevalence of Hypertension in Adults

According to WHO data [17], in 2008, the overall prevalence of raised blood pressure in adults aged 25 and over was around 40%. Across the income groups of countries, the prevalence of raised blood pressure was consistently high, with low, lower middle, and upper middle countries all having rates of around 40%. The prevalence in high income countries was lower at 35%. In the Indian adult population, arterial blood pressure was elevated in 23.1% of males and 22.6% of females compared with Italy (M 28.6% and F 20.6%) and the USA (M 17.0%, F 14.2). Our data in adults [16] show a more serious situation in the tested population, as we found a higher prevalence of stage 1 and stage 2 hypertension, affecting 39.4% of subjects. If we include patients with pre-hypertension, there remain only 24% of people with normal blood pressure levels. The reasons why the prevalence of hypertension appears to be high in the West Bengal population are not currently established. Factors that could be investigated include dietary sodium content, genetic factors, low birth weight, and presence of toxic substances in drinking water.

## 3. Proteinuria and Glomerular Filtration Rate

Urine dipstick is an inexpensive and accessible test for detecting urinary abnormalities [18]. In our overall population, dipstick proteinuria was positive in 7.7% of subjects. In a subsample of high risk subjects with hypertension or urine dipstick proteinuria ≥1+, we found an albumin-creatinine ratio (ACR) of 30–300 and ≥300 mg/g in 23.5% and 2.1%, respectively [16].

The exact Indian prevalence of CKD, defined as GFR < 60 mL/min/1.73 m^2^, is uncertain. Rajapurkar and Dabhi [19], reviewing three published studies carried out in different parts of India, reported a prevalence of CKD ranging from 0.8% to 1.4%. Results of the “Screening and Early Evaluation of Kidney Disease” (SEEK) study have been recently published [20] reporting eGFR < 60 mL/min in 5.9% of subjects (4.3% CKD stage 3, 0.8% CKD stage 4, and 0.8% CKD stage 5). We found a lower prevalence of CKD stages 3 to 5 (4.1% in total) compared to the SEEK study, but a substantially higher prevalence when compared with previous studies, conducted about 10 years ago [19].

## 4. Body Weight Indicators in the Indian Population

The traditional body weight indicator is the body mass index (BMI) [21] expressed as kg/m^2^. We found a median (interquartile range: 1st–3rd) BMI of 19.4 kg/m^2^ (17.4–19.9) in men and 20.8 kg/m^2^ (18.1–23.7) in women, a rather low figure compared to other countries. However, WHO reference values for BMI may not be applied to the Indian population, because in Asia type 2 diabetes and cardiovascular morbidity may be associated with BMI values below the traditional cutoff point of 25 kg/m^2^ that defines overweight in western countries. New BMI categorization by using cutoffs identified for Asians has been proposed [22]. With the more conservative Asian reference values, the combined prevalence of overweight and obesity in our sample population is about 18% and 30% for men and women, respectively.

Waist-to-hip ratio and waist-to-height ratio are anthropometric measures of abdominal adiposity. Independently of BMI, they are considered significant prognostic factors for all-cause and cardiovascular mortality in normal weight people [23] and in CKD patients [24]. These measures are mainly dependent on waist circumference, with very different cutoffs for increased cardiovascular risk between genders and ethnicity. In particular, in western countries, waist circumference should be lower than 102 cm in men and 88 cm in women, while for Asian Americans cutoff points are significantly lower at 90 cm and 80 cm, respectively [25]. In our adult study [16], waist circumference was significantly smaller and similar between genders: men 75 cm and women 73 cm. Keeping in mind these figures, results of waist-to-hip and waist-to-height ratios should be considered with particular attention. Western women with waist-to-hip ratio above 0.8 and men with waist-to-hip ratio above 1.0 are considered at increased health risk. Surprisingly, our female population had a mean waist-to-hip ratio of 0.88 implying a significant health risk despite a small waist circumference. This is due to a relatively narrow hip circumference, which could be possibly related to muscle wasting or to anatomic configuration. We thus proposed that caution should be used in applying the waist-to-hip ratio health risk assessment in the West Bengali female population [16].

Waist-to-height ratio is another screening tool for detecting cardiometabolic risk factors, representing a simple measure valid for men and women of all ethnic groups [26, 27]. In our Indian adults cohort, we found that waist-to-height ratio was strongly related to hypertension. In addition, it was the only anthropometric indicator independently related to the development of proteinuria [16]. Thus, it appears that in the northeastern Indian population the waist-to-hip ratio is poorly reliable, while the waist-to-height ratio is related to both hypertension and proteinuria.

*Rural versus Urban Environment, Hypertension, and Renal Function.* We found an independent association with hypertension for two other parameters: proteinuria and screening setting (rural versus urban) [16]. In particular, living in a rural area reduced the risk of hypertension. This is a relevant finding, although at the moment the reasons for the observed differences remain speculative. The rural setting was also associated with a higher eGFR, after correction for BMI, proteinuria, and hypertension, all factors associated in our population with reduced kidney function, similarly to high income countries [16]. This association has been already reported in India indicating that hypertension is present in 25% urban and 10% rural subjects [28].

## 5. Hypertension in Children

Childhood hypertension is underestimated in low income countries, although it is an established predictor of adult hypertension and organ damage [29]. We studied the prevalence of arterial hypertension as well as the relationship between blood pressure values and weight class or urinary abnormalities in 1176 children (aged 5 to 11 years) in the same area where the adult screening was carried out [15]. The majority of children (75%) were underweight and 5.2% had systolic and/or diastolic BP values higher or equal to the 95th percentile (standard US tables). Hypertension had a relatively high prevalence both in underweight children (4.3%) and in normal weight children (6.9%) and significantly increased in the rare overweight children (4/20 children, or 20%). Despite the presence of hypertension, urinary abnormalities were not different from normal subjects (microhematuria in 8.5% of hypertensive children versus 12.5% in nonhypertensive children). The impact of hypertension found in our study in northeastern Indian children is heavier than in western countries. Jafar et al. [30] also described in south Asian children higher BMI-adjusted blood pressure values compared to children from the US. Remarkably, hypertension was present even in a significant proportion of underweight children [15]. This suggests that in south Asian children, factors different than overweight (e.g., environmental or genetic factors) may play a role in the development of hypertension [31]. In particular, in a population with a great number of underweight individuals, we cannot exclude the fact that some children with arterial blood pressure higher than or equal to the 95th percentile may have had a low body weight at birth and that this fact could have played a role in the genesis of hypertension [32].

In fact, low birth weight and prematurity are the most consistent clinical surrogates for the presence of a low nephron number at birth and are associated with increased risk of hypertension, proteinuria, and kidney disease in adulthood [33]. Indeed, incomplete intrauterine development may affect the maturation of nephrons [34] with consequent development of hypertension because the reduced number of nephrons is associated with a compensatory glomerular hypertrophy and intraglomerular hypertension slowly leading to glomerular sclerosis and the development of systemic hypertension. Whether hypertension in pediatric age in Indian children may influence a hypertensive state and organ damage later in their life remains an unresolved issue. A better understanding of the role of intrauterine development in determining hypertension and renal disease risk is needed: it could help in adopting prevention initiatives with potential significant benefits for the future health and sanitary cost.

## 6. Projects of Future Interventions in the Kolkata, West Bengal Area

**Figure 4:**
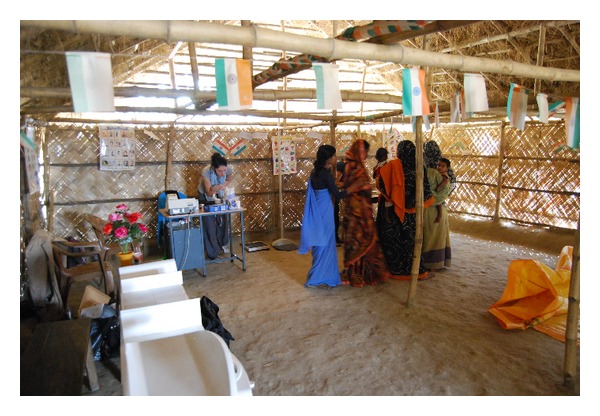
Screening of women of childbearing age.

The collaboration between the institutions that undertook the NCD studies in Kolkata is continuing. There is an ongoing project on the evaluation of hypertension and urinary abnormalities in women of childbearing age (Figure 4). Evaluation of body weight in very young children (less than 2 years old) is also ongoing to investigate the hypothesis of hypertension being influenced by low birth weight. Thus, optimization of maternal health and early childhood nutrition could reduce the global burden of hypertension and kidney disease in the future.

Possible future interventions include the following.

- Evaluation of the prevalence of diabetes mellitus. Glucose levels were determined during the studies on hypertension and CKD, with an unusually high proportion of subjects with increased glucose levels. However, such data have been considered cautiously because we could not be sure that subjects were fasting as required during the screening procedure. A new study could avoid this problem by evaluating glucose levels before and after a prespecified meal.
- Establishment of an outpatient clinic dedicated to the prevention of complications of hypertension and diabetes mellitus, including CKD, through the use of clinical monitoring, dietary and behavioral advice, and the use of low cost medications for the treatment of hypertension (ACE-inhibitors as the first choice) and diabetes mellitus (metformin as the first choice).

## 7. Conclusions

We believe that the information provided by our studies indicates that educational programs should be started with the aim of improving modifiable risk factors (i.e., salt intake, water springs decontamination, nutrition during pregnancy, and so on) that contribute to hypertension and CKD. Dans et al. [4] suggested that surveillance of key modifiable risk factors is needed to monitor the magnitude of the NCD phenomenon and to study the effects of interventions. A united stand against chronic NCDs is warranted, because “inaction will affect millions of lives—often, the lives of those who have the least” [4]. Indeed, prevention would be the most suitable alternative given the economic cost of therapies unsustainable for the poorest countries.

In a setting with limited access to medical care, screening for hypertension and diabetes could be an important initiative for uncovering diseases with preventable complications. Usefulness and cost-effectiveness of urinalysis are debated [35, 36], and the American Association of Pediatrics no longer recommends a screening urinalysis for children. Urine dipstick is inexpensive but probably cost-ineffective. In screening children, Sekhar et al. [36] found 1 case of CKD per 800 screened, with a cost of 2780 US dollars per case diagnosed. Kaplan et al. [35] proposed that a single screening dipstick urinalysis should be obtained at school entry age in all asymptomatic children. It is not known if these suggestions might also apply to the Asian population considering the higher prevalence of uncontrolled diabetes and hypertension. In adults, for early diagnosis and prevention of CKD, our experience suggests that a screening based on a diagnostic algorithm could be a reasonable approach: first, test for the presence of hypertension and hyperglycemia; second, in subjects with abnormal results, test for albuminuria; third, determine serum creatinine levels and eGFR (estimated glomerular filtration rate). Care of end stage kidney disease is very expensive and in many circumstances unaffordable, while early intervention may retard the progression of kidney disease. The medical community should dedicate more efforts to early diagnosis of NCDs, in particular, hypertension, diabetes, and CKD, an increasing public health issue.
